# Faecal bile acids are natural ligands of the mouse accessory olfactory system

**DOI:** 10.1038/ncomms11936

**Published:** 2016-06-21

**Authors:** Wayne I. Doyle, Jordan A. Dinser, Hillary L. Cansler, Xingjian Zhang, Daniel D. Dinh, Natasha S. Browder, Ian M. Riddington, Julian P. Meeks

**Affiliations:** 1 Department of Neuroscience, The University of Texas Southwestern Medical Center, 5323 Harry Hines Boulevard, Dallas, Texas 75390, USA; 2 Neuroscience Graduate Program, The University of Texas, Southwestern Graduate School of Biomedical Sciences, 5323 Harry Hines Boulevard, Dallas, Texas 75390, USA; 3 Department of Chemistry, The University of Texas, 120 Inner Campus Drive, Austin, Texas 78712, USA

## Abstract

The accessory olfactory system (AOS) guides behaviours that are important for survival and reproduction, but understanding of AOS function is limited by a lack of identified natural ligands. Here we report that mouse faeces are a robust source of AOS chemosignals and identify bile acids as a class of natural AOS ligands. Single-unit electrophysiological recordings from accessory olfactory bulb neurons in *ex vivo* preparations show that AOS neurons are strongly and selectively activated by peripheral stimulation with mouse faecal extracts. Faecal extracts contain several unconjugated bile acids that cause concentration-dependent neuronal activity in the AOS. Many AOS neurons respond selectively to bile acids that are variably excreted in male and female mouse faeces, and others respond to bile acids absent in mouse faeces. These results identify faeces as a natural source of AOS information, and suggest that bile acids may be mammalian pheromones and kairomones.

## Abstract (web summary)

The accessory olfactory system (AOS) processes social chemosensory information and guides behaviors that are important for survival and reproduction in mammals. Here the authors report that mouse feces are a source of AOS neuronal activity and identify unconjugated bile acids in feces as a class of natural AOS ligands.

## Graphical abstract

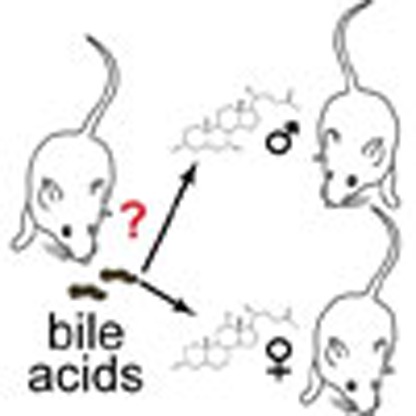

Social communication between most mammals relies heavily on olfaction. Terrestrial mammals employ multiple olfactory pathways for social communication, including the accessory olfactory system (AOS, also called the vomeronasal system)^1^. The AOS processes social chemosensory information from conspecifics (via pheromones) and heterospecifics (via kairomones), and is important for many behaviours, including mating, territorial aggression, pregnancy maintenance and predator avoidance^2^,^3^,^4^,^5^,^6^. A major barrier to understanding AOS function has been lack of knowledge about the system's full complement of natural ligands.

Most of the known AOS ligands were identified by screening excretions from mice and mouse predators^6^,^7^,^8^,^9^,^10^,^11^,^12^,^13^,^14^,^15^. Discovering new natural AOS ligands has proven to be difficult, in large part due to technical barriers to recording the activity of AOS neurons that we and others have recently overcome^9^,^12^,^16^,^17^,^18^,^19^,^20^,^21^,^22^. AOS sensory signalling begins in the vomeronasal organ (VNO), where vomeronasal sensory neurons (VSNs) detect ligands through the expression of just one or two G protein-coupled vomeronasal receptors (VRs)^23^,^24^,^25^,^26^,^27^ or formyl peptide receptors^11^,^12^. VSNs project their axons to the accessory olfactory bulb (AOB), the first and only major dedicated neural circuit for information processing in the AOS^1^,^22^,^28^,^29^. VSNs are rather noisy signal detectors^17^,^30^ but their downstream synaptic targets, the AOB mitral cells (MCs), demonstrate high signal/noise ratios by virtue of a high degree of synaptic convergence from many VSNs expressing the same VR^22^,^28^,^31^,^32^. We used an *ex vivo* preparation of the AOS (ref. 33) that makes use of this network feature in a screen aimed at determining whether faeces contained novel AOS ligands.

There are two main reasons why we investigated faeces as a potential source of AOS activation. First, it has long been known that soiled cage bedding is one of the most potent activators of AOS activity^20^,^34^. Bedding contains a mixture of mouse excretions, the most abundant of which are urine and faeces. Urine, currently the best-studied source of AOS ligands, contains a number of unique ligands including sulfated steroids and major urinary proteins^9^,^10^,^12^,^15^,^17^, but mammalian faecal chemosignals have not yet been systematically investigated. Second, molecular components of faeces vary across many biological states, including sex and species^35^,^36^,^37^,^38^, and information gleaned from faecal constituents might regulate animal behaviour.

We report that faeces are a robust source of AOS ligands that are largely distinct from urinary ligands. The most prominent active components of mouse faeces are bile acids and AOS neurons discriminate between bile acids that vary with sex and species. The discovery of bile acids as natural AOS ligands derived from faeces reveals an external link between the gut and brain that may inform mouse social and reproductive behaviours.

## Results

### VNO stimulation with female faeces activates AOB neurons

**Figure 1:**
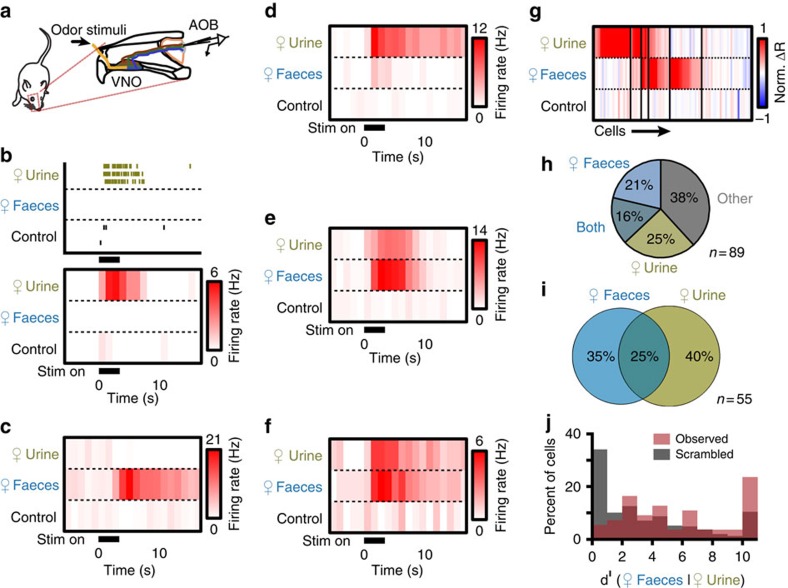
Female faecal extracts activate the AOS. (**a**) Overview of the AOS and the *ex vivo* preparation. (**b**) Example single-unit recording of a male mouse AOB neuron that responded to female mouse urine. Top is a raster plot, bottom is an average peristimulus time histogram (PSTH) from the same cell. (**c**) An AOB neuron exclusively responsive to female faecal extracts. (**d**) A neuron that selectively responded to urine. (**e**) Neuron that selectively responded to faeces. (**f**) A cell that responded equally to both urine and faeces. (**g**) Heat map of normalized change in firing rate (Norm. Δ*R*) following VNO stimulation with female urine or faeces. Thin black lines indicate divisions between clusters (89 cells from 56 animals). (**h**) Percentage of cells that responded exclusively to urine, exclusively to faeces extracts, to both urine and faeces extracts, or to other compounds in the stimulus battery (89 cells from 56 animals). (**i**) Venn diagram of response overlap for the pool of neurons that responded to urine and/or faeces (55 cells from 40 animals). (**j**) Histogram showing the discriminability index (*d*^ı^) for the observed AOB neuron population (red) compared with the mean of 100,000 scrambled populations (grey; 55 cells from 40 animals).

We investigated the potential for faecal chemosignals to activate the AOS using single-cell electrophysiological recordings from *ex vivo* preparations that maintain functional connectivity between the VNO and AOB (Fig. 1a)^28^,^33^,^39^. We developed an aqueous extraction protocol to isolate water-soluble chemosignals from female BALB/cJ faeces (Methods section). We chose an aqueous extraction because AOS ligands must dissolve in the aqueous nasal mucus to be carried from the nares into the VNO *in vivo*.

We delivered dilute BALB/cJ female faecal extract to the VNO of *ex vivo* preparations from male B6D2F1/J mice while making single-unit electrophysiological recordings from downstream AOB MCs (Fig. 1a,b). As a positive control, we stimulated the VNO with 100-fold diluted BALB/cJ female urine, a robust source of AOS chemosignals (Fig. 1b)^15^,^18^,^20^,^40^. Diluted faecal extracts (300-fold) produced robust, but selective activity in AOB MCs (Fig. 1b–f). We observed MCs that responded exclusively to urine or faeces (Fig. 1b,c), MCs that selectively responded to urine or faeces (Fig. 1d,e) and MCs that responded to both (Fig. 1e). Across the population, 21.4% of AOB MCs responded selectively to 300-fold diluted BALB/cJ female faeces, nearly equivalent to the selective activation by 100-fold diluted BALB/cJ female urine (24.7%; 89 cells from 56 animals Fig. 1g,h). 15.7% of MCs responded nonselectively to faecal extracts and urine, while 38.2% of MCs did not respond to either of these stimuli, but did respond to other chemosignals in our stimulus battery (Fig. 1g–i). Among MCs that responded to urine and/or faeces, 34.5% of the cells responded selectively to faeces, 40% responded selectively to urine and 25.5% responded to both (55 cells from 40 animals Fig. 1i). In separate *in vivo* experiments, we introduced male mice to BALB/cJ female soiled bedding or to clean bedding mixed with BALB/cJ female urine or faecal extracts. Expression of the immediate-early gene Fos was increased in the AOBs of these mice, confirming that faecal chemosignals activate the AOS *in vivo* (Supplementary Fig. 1).

Faecal extracts and urine at these dilutions were equally potent. The apparent selectivity of many MCs indicated that urine and faeces produce unique information in the AOS. We quantified the capacity for this information to be used to discriminate urine from faeces by calculating the discriminability index (*d*^ı^) for all MC responses to urine and faeces based on the change in firing rate (Δ*R*) elicited by each (Fig. 1j). The *d*^ı^ values we observed across the MC population showed a strong bias towards high discriminability between urine andz faeces (Fig. 1j). 98.9% of 100,000 simulated MC populations, each with 55 urine and faeces responses chosen at random from our actual data, showed *d*^ı^ distributions that were statistically lower than the observed population. In sum, the strong but differential activation of MCs by faecal and urinary chemosignals indicated that urine and faeces provide unique information to the AOS.

### Polar molecules are a primary source of AOS activity to faeces

**Figure 2:**
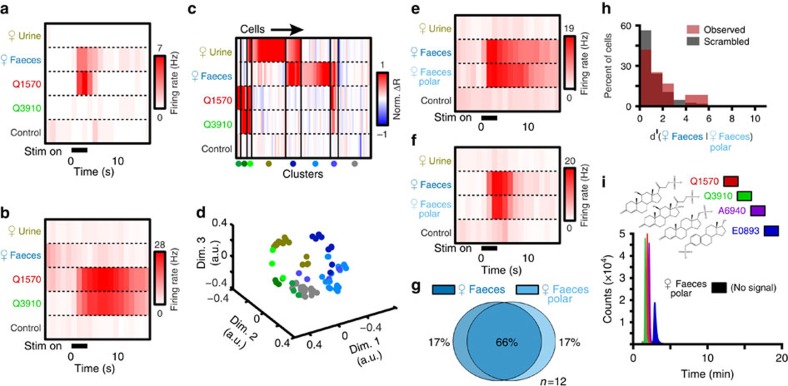
Female faecal extract activity is not due to sulfated steroids. (**a**) Peristimulus time histograms of AOB neuron that responded to 10 μM corticosterone-21-sulfate (Q1570) and 300-fold dilute BALB/cJ female mouse faecal extract. (**b**) Cell that responded to 10 μM Q1570 and 10 μM hydrocorticosterone-21-sulfate (Q3910) but not to female mouse faecal extract. (**c**) Heat map of responses to female mouse urine, faecal extract, Q1570 and Q3910. Thin black lines indicate divisions between clusters (87 cells from 55 animals). (**d**) Multidimensional scaling of the AOB responses to faeces, urine and glucocorticoids. Colours correspond to the clusters in **c**. (**e**,**f**) Two AOB neurons that responded to female mouse faeces (1:300 dilution) and female mouse faeces polar fraction (1:100 dilution). (**g**) Venn diagram showing overlapping responses between female faecal extract and its polar fraction (12 cells from 6 animals). (**h**) Histogram of the *d*^ı^ values for female faeces extract and its polar fraction. Red bars indicate the observed *d*^ı^. Grey bars indicate the average *d*^ı^ of 100,000 shuffled populations. (**i**) Overlaid extracted ion chromatograms (EICs) showing [M-H]- signals for standards: Q3910 ([C21H30O8S-H]^−^, green), Q1570 ([C21H30O7S-H]^−^, red), epitestosterone-17-sulfate (A6940, [C19H28O5S-H]^−^, purple), 17α-estradiol-3-sulfate (E0893, [C18H24O5S-H]^−^, blue) and female faeces (black, not visible).Female faeces polar extract at these [M-H]^−^ values produced no detectable signal.

Sulfated glucocorticoids are known urinary AOS chemosignals that robustly activate the anterior AOB (aAOB)^15^,^28^,^39^. The partial overlap in urine- and faeces-driven aAOB activity suggested that they may share common ligands, so we tested whether urinary sulfated steroids were present in both urine and faeces. Consistent with this hypothesis, we encountered some MCs that responded to corticosterone-21-sulfate (Q1570, 10 μM) and 300-fold diluted faecal extracts (Fig. 2a). Cells that responded to both faecal extracts and glucocorticoids were rarer (5/87 MCs) than MCs responding to glucocorticoids but not faeces (10/87 MCs; Fig. 2b,c). We used clustering algorithms to classify the tuning curves of 87 MCs recorded during VNO stimulation with mouse urine, mouse faecal extracts and two sulfated glucocorticoids (Fig. 2c, 87 cells from 55 animals). Multidimensional scaling, a method that assists in the visualization of multi-factor tuning differences, highlights the approximate magnitude of tuning differences between cells in each of the eight identified clusters (Fig. 2d). Though MCs tuned to both faeces and glucocorticoids were rare, these results suggested that glucocorticoids may be shared ligands between urine and faeces. Alternatively, glucocorticoids at 10 μM may activate receptors that are also activated by different faecal ligands.

Sulfated steroids were originally discovered in the polar fraction of chloroform:methanol extractions of mouse urine^15^,^41^. To determine whether faecal AOS ligands were also polar, we compared MC tuning to polar fractions of faecal chloroform:methanol extractions to the whole-faecal extracts. Polar faecal extracts strongly co-activated faeces-tuned MCs in the aAOB (Fig. 2e,f). Of the 12 cells tested with the whole extracts and polar fraction, 66.6% of cells responsive to whole-faecal extract also responded to the polar fraction (12 cells from 6 animals Fig. 2g). Furthermore, discriminability analysis showed that tuning to whole-faecal extract and its polar fraction was similar to tuning curves chosen randomly (statistically significant in only 17.4% of 100,000 simulated MC populations; Fig. 2h). Thus, the majority of faecal extract activity in the aAOB was caused by polar molecules. To determine whether these polar molecules contained sulfated steroids, we performed high resolution, accurate-mass liquid chromatography–mass spectrometry (LC–MS) on the faecal polar fractions. We did not detect appreciable levels of sulfated glucocorticoids, androgens or estrogens (Fig. 2i), indicating that sulfated steroids are not a major source of faeces-driven activity in the aAOB.

### The active AOS ligands in female faecal extracts are bile acids

**Figure 3:**
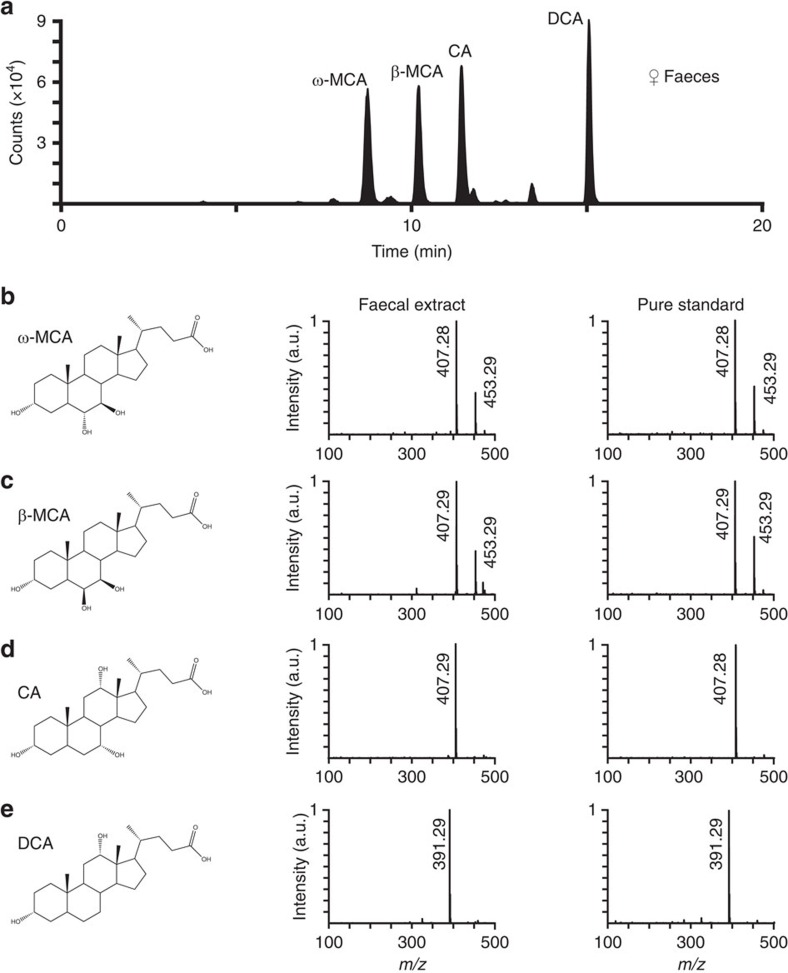
Female faecal extracts contain unconjugated bile acids. (**a**) Extracted ion chromatograms (EICs) showing [M-H]^−^ signals for C_24_H_40_O_4_ (deoxycholic acid) and C_24_H_40_O_5_ (cholic and muricholic acids) in female mouse faecal extract. Identified compounds are indicated above each peak. (**b**–**e**) Mass spectra of peaks for bile acid standards and faecal extracts. Peak times refer to times from **a**. (**b**,**c**) ω-muricholic acid (ω-MCA, (**b**) at 8.8 min and β-MCA (**c**) at 10.2 min. Peaks at *m*/*z* 453.29 indicate the presence of formate adducts. (**d**) CA at 11.4 min. (**e**) DCA at 15.0 min.

LC–MS revealed the presence of distinct, abundant peaks for molecules with mass-to-charge (*m*/*z*) ratios consistent with unconjugated bile acids (Fig. 3). Bile acids are polar sterols vital for lipid and vitamin absorption in vertebrates, and are known to be excreted in faeces^42^,^43^,^44^. We compared the spectra and elution times of several pure bile acids, finding that the most abundant molecules within the female faeces polar fraction were ω-muricholic acid (ω-MCA, Fig. 3b), β-MCA (Fig. 3c), cholic acid (CA, Fig. 3d) and deoxycholic acid (DCA, Fig. 3e). These results suggested that bile acids may be the active polar ligands in faecal extracts.

**Figure 4:**
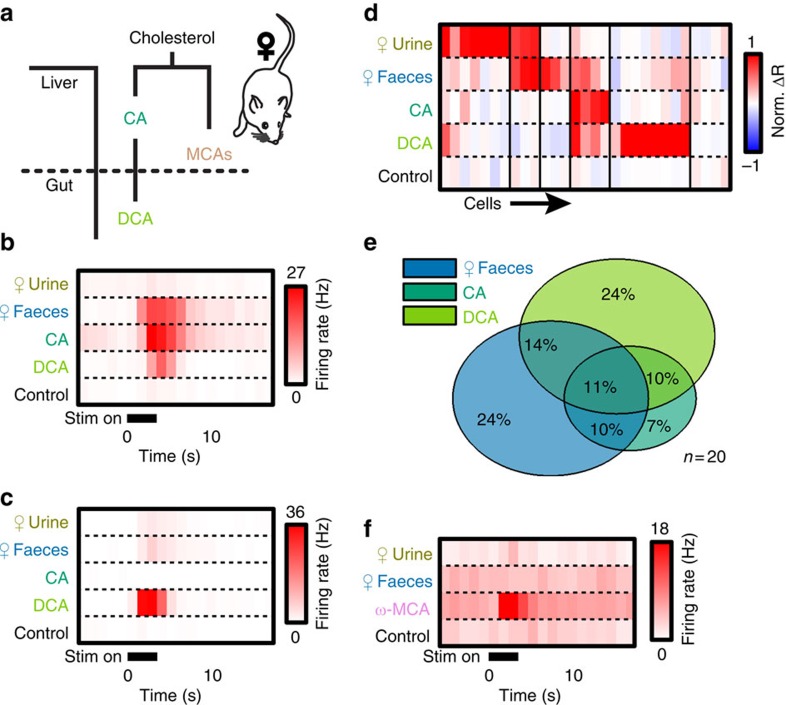
The AOS is activated by BAs present in female mouse faeces. (**a**) Simplified synthesis pathway for CA, DCA and MCAs in the female mouse. (**b**) Peristimulus time histograms of a cell that responded to faeces, CA and DCA. (**c**) A cell that responded to DCA but not to CA. (**d**) Heat map of AOB responses to urine, faeces, CA and DCA. Thin black lines indicate divisions between clusters (29 cells from 14 animals). (**e**) Venn diagram of responses to faeces, CA and DCA (20 cells from 11 animals). (**f**) An AOB neuron that responded to the rodent-specific bile acid ω-MCA.

We initially sought to determine whether the most abundant bile acids indicated by LC–MS were natural AOS ligands. CA is a primary bile acid produced by the liver, whereas DCA is a secondary bile acid produced by gut microbes via CA dehydroxylation (Fig. 4a)^45^,^46^,^47^. CA and DCA were delivered to the VNO at 10 μM, a concentration that for pure sulfated steroids produced strong but selective activation of AOS neurons^21^,^28^. Both CA and DCA produced robust responses within the aAOB, and MCs exhibited specific tuning to these ligands (29 cells from 23 animals, Fig. 4b–d). Many MCs that responded to 10 μM CA or DCA were co-activated by 300-fold diluted female faecal extracts. Not all MCs that responded to 10 μM CA or DCA also responded to the diluted faecal extracts, suggesting that, at this dilution, the effective concentration of these ligands was <10 μM. Among the 20 MCs responsive to faeces, CA or DCA, 10% responded exclusively to CA, 35% responded exclusively to DCA and 35% responded exclusively to female faeces. Fifteen percent of the cells examined responded to CA, DCA, and female faeces (Fig. 4e). These results indicate that though CA and DCA are structurally similar, the AOS is capable of differentiating between these two compounds at 10 μM. Responses to ω-MCA, a rodent-specific bile acid, were also observed, but were much rarer than for CA and DCA (Fig. 4f). A mixture of four pure bile acids, all at 1 mM, delivered directly to the nares *in vivo* effectively induced Fos expression in aAOB neurons (Supplementary Fig. 2), confirming that these natural ligands activate the AOB following dilution and transport via the nasal mucus.

**Figure 5:**
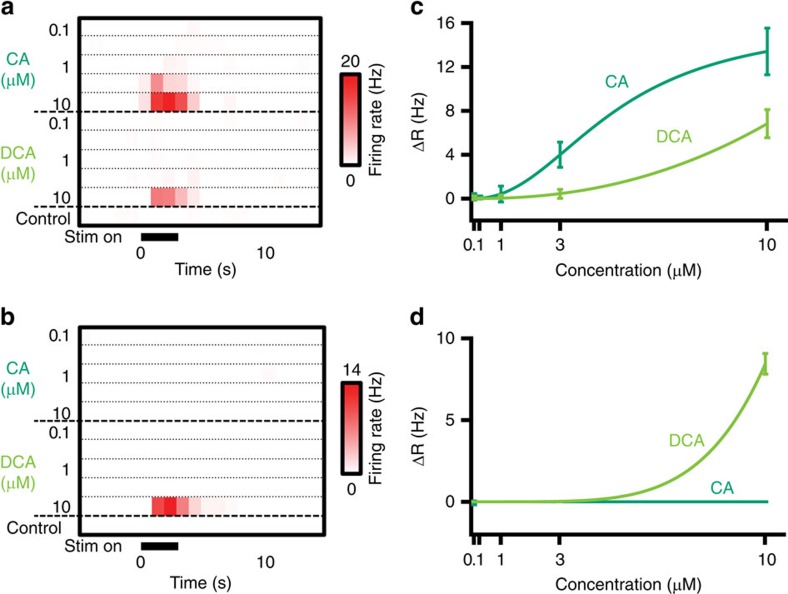
Dose-responses to CA and DCA. (**a**) Peristimulus time histograms (PSTH) of an AOB neuron that responded to both CA and DCA across a range of concentrations. (**b**) PSTH of a cell that responded to DCA only at 10 μM. (**c**,**d**) Dose response curves for the cells in **a** and **c**, respectively (four cells from three animals). Error bars reflect across-trial s.e.m.

To directly investigate the concentrations at which CA and DCA were active, we evaluated the concentration-dependence of MC responses to DCA and CA in a subset of *ex vivo* experiments (four cells from three animals, Fig. 5). Neurons that responded to both CA and DCA at 10 μM showed strong selectivity for CA at 3 μM and ceased to respond below 1 μM (three cells from three animals Fig. 5a,c). A cell that was exclusively tuned to DCA failed to respond to either bile acid at concentrations below 10 μM (Fig. 5b,d). Thus, bile acids indeed produce selective, concentration-dependent MC activation, consistent with selective activation of different VRs by bile acids.

### AOB neurons discriminate between male and female faeces

**Figure 6:**
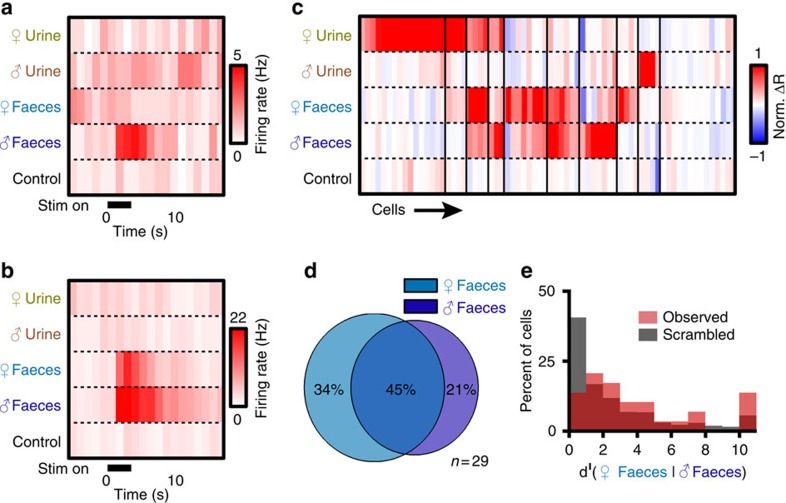
AOB neurons discriminate between male and female faecal extracts. (**a**) An AOB neuron that responded exclusively to male mouse faecal extract. (**b**) An AOB neuron that responded to both female and male faeces. (**c**) Heat map of neuronal responses to female and male urine and faeces. Thin black lines indicate cluster divisions (70 cells from 44 animals). (**d**) Venn diagram of AOB responses to female and male faeces (29 cells from 23 animals). (**e**) Histogram of *d*^ı^ values for male and female mouse faeces. Red bars indicate the observed *d*^ı^ values. Grey bars indicate the average *d*^ı^ for 100,000 shuffled populations (29 cells from 23 animals).

Animal secretions (for example, tears and urine) contain sex-specific AOS cues^4^,^7^,^34^,^48^, and previous studies indicated that the bile acid pool also varies with sex^35^. Therefore, we investigated whether male and female faeces differentially activate the AOS. We delivered 300-fold diluted faecal extracts from BALB/cJ males and females to male *ex vivo* preparations, and recorded sex-specific activation of AOB MCs (Fig. 6). Many MCs responded selectively to faecal extracts from a specific sex (Fig. 6a) and others responded to faecal extracts of both sexes (Fig. 6b). We classified MC tuning curves to male and female urine and faeces using clustering algorithms, revealing variable tuning patterns (70 cells from 44 animals Fig. 6c). For the 29 cells that responded to male or female faeces, 34.5% of those cells responded selectively to female faeces, 20.7% responded selectively to male faeces and 44.8% responded nonselectively to both (Fig. 6d). We quantified the discriminability of male and female faeces by AOB MCs, finding that, as a population, sex discrimination based on faeces was lower than between female urine and faeces, but that the presence of many MCs with high *d*^ı^ values was significant (67.9% of 100,000 simulated MC populations showed statistically lower discriminability than observed; Fig. 6e). These results indicated that there are sex-specific differences in the concentrations and/or identities of faecal chemosignals.

**Figure 7:**
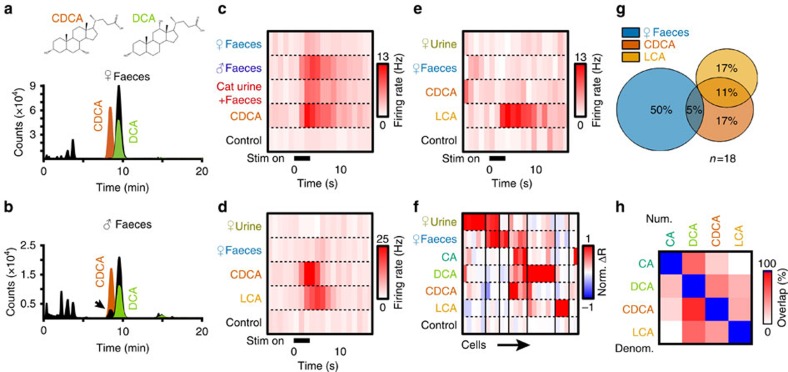
CDCA and LCA acids are detected and discriminated by the AOS. (**a**) EICs showing [M-H]^−^ signals for C_24_H_40_O_3_ (LCA) and C_24_H_40_O_4_ (DCA) for female mouse faecal extract (black). Overlaid are EICs for CDCA (orange) and DCA standards (green). Female faecal extract contains DCA and not CDCA. (**b**) EICs showing [M-H]^−^ signals for C_24_H_40_O_3_ (LCA) and C_24_H_40_O_4_ (DCA) for male fecal extract (black). Overlaid are EICs for CDCA (orange) and DCA standards (green). Male faeces contain both CDCA and DCA. (**c**) An AOB neuron that responded to male mouse faeces, a cat urine+faeces mixture, and CDCA. (**d**) Peristimulus time histograms of a cell that responded to CDCA and its derivative LCA. (**e**) A cell that responded to LCA and not to CDCA. (**f**) Heat map of AOB responses to female mouse urine, female faeces, CA, DCA, CDCA and LCA. Thin black lines indicate divisions between clusters (25 cells from 13 animals). (**g**) Venn diagram of shared responses among female faeces, CDCA and LCA (18 cells from 10 animals. (**h**) Pairwise comparisons of neuronal responsiveness to CA, DCA, CDCA and LCA. Percentages indicate the number of neurons that were co-activated by the row/column pair divided by the total pool of neurons that responded to the row stimulus (25 cells from 13 animals).

To determine whether male and female faeces contained different bile acids, we analysed male and female faecal extract polar fractions by LC–MS (Fig. 7). We found that one major sex-specific difference was the presence of chenodeoxycholic acid (CDCA) in male but not female faecal extracts (Fig. 7a,b). CDCA, like CA, is a primary bile acid produced in the liver, but CDCA and its secondary derivative lithocholic acid (LCA) are most commonly associated with non-rodent species^36^,^37^,^38^. CDCA and LCA levels are low in most mice, which is thought to be due to rapid conversion of CDCA to MCAs in mice and other rodents^49^. The presence of CDCA in BALB/cJ male faeces indicated that the detection of CDCA may be one mechanism by which AOS neurons could discriminate between BALB/cJ males and females. Moreover, since CDCA is present in the faeces of many heterospecifics, this bile acid may also contribute in a combinatorial fashion to other chemosensory discrimination tasks.

### The AOB discriminates conspecific from heterospecific bile acids

We investigated AOB MC tuning to CDCA, mouse faeces and cat urine that had been collected after being in direct contact with faeces (Methods section). We found individual MCs that responded to 10 μM CDCA, male faeces and 100-fold dilutions of the cat urine+faeces sample (Fig. 7c). We also investigated AOB neuronal tuning to LCA, which was not detectable in mouse faeces, but is present in other species^36^,^37^,^38^. We encountered MCs that were responsive to both 10 μM CDCA and 10 μM LCA (Fig. 7d), as well as cells that were exclusively responsive to 10 μM LCA (Fig. 7e). Similar results were observed in volumetric VSN Ca^2+^ imaging experiments (Supplementary Fig. 3). We evaluated MC tuning to CA, DCA, CDCA and LCA for 25 MCs exposed to all of these ligands using cluster analysis (Fig. 7f). MCs responding to CDCA and/or LCA rarely responded to female mouse faeces (Fig. 7g). Pairwise comparisons of bile acid responsiveness showed that LCA-responsiveness was mutually exclusive with CA responsiveness (Fig. 7h). These results indicate that the AOS discriminates between bile acids present in conspecific and heterospecific faeces.

**Figure 8:**
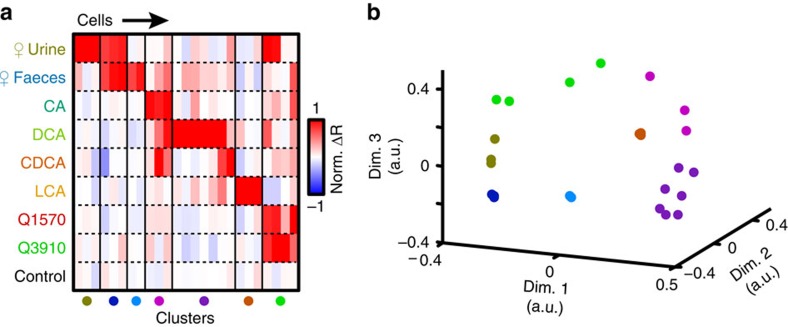
Tuning of AOB neurons to urine, faeces, sulfated glucocorticoids and BAs. (**a**) Heat map of AOB responses to female mouse urine, female faeces, CA, DCA, LCA, Q1570 and Q3910. Thin black lines indicate cluster divisions (25 cells from 13 animals). (**b**) Multidimensional scaling of the AOB responses to faeces, urine, glucocorticoids and BAs. Colours correspond to the clusters in **a**.

Finally, we investigated MC tuning across all the polar sterols in our stimulus battery, including primary and secondary bile acids and sulfated glucocorticoids (Fig. 8). Cluster analysis revealed rich combinatorial tuning across sterols, but clearly showed that MCs tuned to bile acids were almost completely separable from MCs tuned to sulfated glucocorticoids (Fig. 8a,b). Overall, these results indicated that AOS bile acid tuning is not limited to molecules excreted by conspecifics, and that bile acids produce a complex combinatorial code in AOB MCs, similar to codes produced by sulfated steroids, major urinary proteins, formyl peptides, major histocompatibility proteins and exocrine-secreting gland peptides^7^,^8^,^9^,^10^,^11^,^12^,^13^,^15^,^21^,^28^,^48^,^50^.

## Discussion

Despite two decades of research since the discovery of VRs (refs 6, 7, 8, 9, 10, 11, 12, 15, 23, 24, 26, 27, 48), our understanding of the repertoire of ligands for this behaviourally relevant neural pathway remains incomplete. Technical improvements have made it possible to conduct AOS ligand screens using VSNs as bioassays^6^,^9^,^10^,^15^,^20^,^40^,^50^. However, VSNs are notoriously noisy^17^,^28^,^30^,^51^ and require extensive controls to avoid false positive results. AOB MCs, in contrast, have dramatically improved signal/noise ratios by virtue of synaptic pooling/averaging^28^. In this study, we utilized MC recordings from the *ex vivo* preparation (which maintains functional connectivity between the VNO and AOB) as the platform of a screen for AOS ligands.

Simple aqueous extraction procedures isolate AOS ligands from BALB/cJ female mouse faeces that, at 300-fold dilutions, produce equivalent AOB neuronal activity to 100-fold diluted BALB/cJ female mouse urine. This indicates that mouse faeces are rich in AOS ligands, and that the activity stimulated by these ligands is roughly equivalent to mouse urine, which is currently the best-known source of AOS ligands^9^,^10^,^12^,^15^,^17^. The concentration-related differences in stimulus potency are likely related to the relative dryness of raw faeces compared with mouse urine and the specific ratio of faeces:water used in extractions. That said, faeces are dry in the natural environment, so faecal ligands are likely to be highly concentrated before dissolution in nasal mucus. *In vivo* studies confirmed that dilute faecal extracts produced AOB activation similar to soiled bedding, further indicating that the faecal molecules in these aqueous extracts are biologically active. AOB MCs readily discriminate urine from faeces, indicating that faecal chemosignals produce unique information in the AOS.

The discovery of faeces as a source of distinct AOS ligands is noteworthy for several reasons. First, faeces are plentiful in natural environments and can persist for long periods of time. Second, faeces contain a biochemical readout of internal digestion through molecules that are distinct from other known classes of AOS ligands, such as urine and tears. Third, there is increasing evidence that internal gut–brain feedback influences many neurobiological processes^52^. Our data reveal that faecal chemosignals also activate sensory processing neurons in the AOS, establishing a link between the gut physiology of an animal and its own brain (that is, external gut–brain feedback) or another animal's brain (that is, as a source of pheromones or kairomones).

MC recordings revealed that faecal chemosignals strongly activate the aAOB, which receives selective innervation from VSNs that express members of the V1R subfamily of VRs and certain formyl peptide receptors^31^,^32^,^53^,^54^. Previous studies have strongly implicated V1Rs and the aAOB in the detection of low molecular weight ligands, including urinary sulfated steroids^28^,^39^. Urine- and faeces-responsive AOB were physically collocated in the aAOB, and many aAOB MCs were activated by both faeces and urine. Taken together, these indicate that AOB neurons process information about urinary and faecal chemosignals in the same V1R-receiving subcircuit, and that these two distinct natural ligand sources produce partially overlapping information.

The majority of faeces-driven AOS activity was maintained in the polar fraction of faecal extracts. A small population of MCs was co-activated by sulfated glucocorticoids and faecal extracts, suggesting that sulfated steroids may be faecal AOS ligands. LC–MS revealed no detectable sulfated steroids in the polar fraction of these extracts, but instead revealed the presence of bile acids, of which CA and DCA were among the most abundant. Bile acids are cholesterol derivatives that are produced in the liver and excreted into the gut, where they aid in the absorption of lipophilic substances and act as signalling molecules for several metabolic processes^55^,^56^,^57^,^58^,^59^. The complement of excreted bile acids in faeces varies across sex and species, and secondary bile acids like DCA depend on dehydroxylation and other transformations by gut microbes^35^,^37^,^46^,^47^,^60^. As such, bile acids, as a class, possess many features that would make them potentially instructive chemosignals. That these molecules could be important chemosignals is not totally unprecedented, as bile acids are known to act as pheromones in fishes^61^,^62^,^63^. We found robust, selective, concentration-dependent activation of MCs by DCA and CA, and confirmed that a mixture of bile acids generates robust AOB following *in vivo* exposure. These data confirm that faecal bile acids are natural AOS ligands, and reveal that bile acid chemosignaling is conserved between fishes and mammals.

We tested the capacity of bile acids to serve as readouts of biologically relevant features (for example, sex, species and so on). First, we observed MCs that can discriminate between CA, a primary bile acid produced in the liver, and DCA, which is produced by gut microbes^47^,^64^. This indicates that gut microbiota influence AOS activation by faeces. Next, we investigated whether faecal ligands varied with sex. We compared MC tuning for BALB/cJ male and female faecal extracts and found that many MCs can discriminate between male and female faeces at equal dilutions. Although there may be many ligands that underlie this effect, we confirmed by LC–MS that the primary bile acid CDCA, which was undetectable in female faecal extracts, was present in male faecal extracts. Many MCs that were activated by 10 μM CDCA also responded to male, but not female, faeces. This indicates that CDCA is one ligand that varies with sex, and suggests that CDCA signalling may underlie sex discrimination from faeces.

Whereas CA and DCA are present in a number of species and in both sexes, CDCA is rapidly converted to MCAs in rodents^49^. In other mammals, including mouse predators, CDCA is more prominent^36^,^37^,^38^. Consistent with these reports, we found that several MCs that responded to CDCA and male faeces also responded to cat faeces. Interestingly, we found that AOB responses to rodent-specific MCAs were quite sparse. In contrast, LCA, a secondary bile acid and CDCA metabolite that is absent in mice but present in other mammalian species, including humans, caused robust, highly selective MC activation at 10 μM. The biological significance of these differentially tuned MC populations remains to be discovered, but these data indicate that conspecific and heterospecific bile acids generate distinct signals in AOB neurons. Although additional studies will be necessary to test this hypothesis, these observations suggest that bile acids may also serve as kairomones (cross-species chemosensory cues that benefit the receiver).

MC tuning across bile acids and sulfated glucocorticoids shows that bile acids, similar to other classes of AOS ligands, activate the AOB with a complex combinatorial code^28^,^39^,^40^,^50^. The capacity of the AOS to distinguish between individual bile acids that differ across biological states (for example, sex, species, etc.) indicates that these ligands may drive state-specific behaviours. Future studies that investigate the behavioural impact of the combinations of bile acids found in natural samples will be necessary to identify these state-specific behaviours. It is worth noting that common themes of sex, species and other biological state-related differences exist across AOS ligand classes, and in nearly all cases, ligands that vary with biological state have been shown to influence mouse behaviours^4^,^6^,^8^,^9^,^40^,^48^,^50^,^65^. The apparent redundancy of sex, species and other biologically relevant information across AOS ligand classes raises the question: what is the biological benefit of processing all this redundant information? The answer is likely to require detailed investigation of the specific social contexts in which the information is encountered.

In summary, we have demonstrated that faeces are a natural source of AOS chemosignals, and that bile acids are a prominent class of AOS ligands. AOB MCs readily discriminate between components of faeces and urine, and between male and female mouse faeces. MCs discriminate between individual bile acids that vary with sex, species and gut microbiota. This discovery reveals a signalling pathway linking gut physiology to the brain, and opens up new avenues for studying the impacts of gut metabolism on mammalian behaviour.

## Methods

### Animals

Electrophysiology experiments were performed with male C57Bl/6J and B6DF2F1/J mice between 6 and 15 weeks of age. VNO imaging experiments were performed with *Omp*^*tm4(cre)Mom*^/J knock-in mice (*OMP-Cre* mice; Jackson Laboratory Stock #006668)^66^ mated to *Gt(ROSA)26Sor*^*tm38(CAG-GCaMP3)Hze*^/J mice (Ai38 mice; Jackson Laboratory Stock # 014538)^67^. Faeces and urine were collected from male and female BALB/cJ mice aged 6–13 weeks. Mice were provided food and water *ad libitum* and kept on a 12:12 light:dark cycle. Mice were fed 16–18% protein by weight chow (Harlan Teklad Global Rodent Diet). All procedures were approved by the University of Texas Southwestern Medical Center Institutional Animal Care and Use Committee and follow guidelines from the National Institutes of Health.

### Stimuli and reagents

All reagents were purchased from Sigma-Aldrich (St Louis, MO, USA) unless otherwise specified. Stimuli were dissolved to their final concentrations in Ringer's solution containing (in mM): 115 NaCl, 5 KCl, 2 CaCl_2_, 2 MgCl_2_, 25 NaHCO_3_, 10 HEPES and 10 glucose. Mouse urine and faeces were collected from 20 female and 10 male BALB/cJ mice for 2–6 weeks. Mice were suspended in a wire-bottomed cage over liquid nitrogen for 4–8 h per day. At the end of collection, frozen urine and faeces were stored at −80 °C until extraction. Urine was extracted as previously described^15^,^28^. Urine was thawed, pooled and centrifuged at 80 *g* for 2 min. The supernatant was removed and filtered through a 0.22-μM filter, aliquoted and stored at −80°C until use. For all physiology experiments, urine was diluted 1:100 in Ringer's solution.

Faecal particles were diluted 1:10 (w:v) in dH_2_O, homogenized and left overnight at 4 °C on an orbital shaker. The suspension was homogenized again and centrifuged at 2,400*g* for 10 min and 2,800*g* for 30 min. The supernatant was filtered through a 0.22-μM filter, aliquoted and stored at −80 °C. Polar faecal extracts were obtained from the aqueous phase of Bligh-Dyer (methanol:chloroform:water) extractions of whole-faecal extracts. Whole-faecal extract was extracted with chloroform:methanol:water at a ratio of 2:2:1.8. Whole extracts of mouse faeces were used as stimuli at 1:300 and polar extracts were used at 1:100.

Cat urine/faeces was purchased from BioreclamationIVT (Westbury, NY, USA) and used at a dilution of 1:100. As indicated by the vendor, cat urine was collected after it had been in direct contact with cat faeces, thus allowing for direct mixing between the two substances.

All sulfated steroids were purchased from Steraloids, Inc. (Newport, RI, USA). Steroids used included the epitestosterone-17-sulfate (A6940) and testosterone sulfate (A7010), 17α-estradiol-3-sulfate (E0893), and corticosterone-21-sulfate (Q1570) and hydrocortisone-21-sulfate (Q3910). Stock solutions (20 mM) of A6940, A7010, E0893 and Q3910 were prepared in methanol. 20 mM Q1570 stock was prepared in dH_2_O. All sulfated steroids were diluted to 10 μM for experiments.

CA, DCA, CDCA, LCA, α-MCA, β-MCA and ω-MCA were purchased from Sigma-Aldrich. Bile acid stocks (20 mM) were prepared in methanol and were diluted to 10 μM for experiments.

### *Ex vivo* preparation

Mice were anaesthetized with isofluorane and decapitated into ice-cold artificial cerebrospinal fluid (aCSF) containing (in mM): 125 NaCl, 2.5 KCl, 2 CaCl_2_, 1 MgCl_2_, 25 NaHCO_3_, 1.25 NaH_2_PO_4_, 25 glucose, 3 *myo*-inositol, 2 sodium pyruvate and 0.5 sodium ascorbate. An additional 9 mM of MgCl_2_ was added to the dissection aCSF to limit excitotoxicity. Half of the mouse skull, from the snout to the olfactory bulbs, was dissected out and adhered to a small plastic plank with tissue adhesive. The plastic plank was inserted into a custom-built dissection chamber where it was superfused with room temperature aCSF. A secondary microdissection was performed to expose the vomeronasal nerves and AOBs. A thin 0.0045 inch cannula (A-M Systems, Carlsborg, WA, USA) was placed into the VNO with a steady stream of fresh VNO Ringer's. The cannula was subsequently used for the delivery of stimulus batteries through the use of a pressurized system (AutoMate Scientific, Berkeley, CA, USA). Stimulus batteries were delivered to the VNO in a randomized, interleaved manner. Stimuli were delivered for 3–5 s, and each cell was exposed to no fewer than three stimulus repeats^28^.

### Electrophysiology

Extracellular recordings were made from the AOB through glass electrodes with resistances between 1.5 and 8 MΩs. Electrodes were filled with 0.22 μm filtered aCSF and advanced into the AOB by a micromanipulator (Siskiyou Corporation, Grants Pass, OR, USA). All recordings were made from neurons generating large positive spikes that were located between 94 and 351 μm from the AOB surface, characteristic of MCs in the AOB external cellular layer^28^. Signals were amplified through a Cornerstone BVC-700A amplifier (Dagan Corporation, Minneapolis, MN, USA), digitized by an analog digital device (National Instruments Corporation, Dallas, TX, USA), and controlled by custom software written in LabVIEW. Recordings were sorted for single-unit activity using custom MATLAB (MathWorks, Inc., Boston, MA, USA) programs^28^,^68^. Cells included in analysis had clearly separable waveforms (evaluated by principal component analysis, spike autocorrelograms and spike crosscorrelograms). All cells included in analysis responded to at least one stimulus and completed at least three complete stimulus trials.

### VNO imaging

We performed Ca^2+^ imaging experiments in mice expressing the genetically encoded Ca^2+^ indicator GCaMP3 in olfactory sensory neurons and VSNs (*OMP-cre*^+/−^, Ai38^+/−^ double transgenics). VNOs were removed from male mice into ice-cold Ringer's solution following deep isofluorane anaesthesia and rapid decapitation^21^. The vomeronasal epithelium was dissected away from adjacent tissue under a dissection microscope (Leica Microsystems, Buffalo Grove, IL, USA), mounted onto a small piece of nitrocellulose paper (Fisher Scientific, Atlanta, GA, USA) and placed in a custom imaging chamber. Live Ca^2+^ imaging of VSNs was performed using a custom objective coupled planar illumination microscope based on earlier models^69^. GCaMP3 fluorescence was recorded during randomized, interleaved stimulus delivery system (Automate Scientific) using custom software^21^,^69^. Image stacks spanning 700 μm (lateral) and 400 μm (axial) were taken once every 2 s. Stimuli were delivered for five consecutive stacks (∼10 s) with 10 stacks between stimulus trials (20 s). No fewer than three stimulus repeats were completed per experiment.

### Mass spectrometry

For female mouse faecal analysis and the initial detection of bile acids, LC was performed as previously described^70^, with minor variations. All experiments were run with an injection volume of 5 μl. Standards were prepared by diluting 5 μl of 5 μg ml^−1^ of the standard with 20 μl of 2:1.8 MeOH:H_2_O. Biological extracts were injected with no dilution. Chromatographic separation was performed on a C18 HPLC column with embedded polar amide groups (Accucore Polar Premium, 2.6 μm particle size, 150 × 2.1 mm). The injected volume was eluted at a flow rate of 0.3 ml min^−1^ using a gradient method where mobile phase A was water and mobile phase B was methanol (Fisher). Both mobile phases contained 0.1% formic acid (Thermo Scientific) and 5 mM ammonium acetate (Fisher). Elution was performed with 60% mobile phase B for 2 min, followed by a gradient increase in mobile phase B to 100% over 18 min. The column was held at 100% mobile phase B for 30 min and then returned to 60% mobile phase B over 3 min followed by a 13-min equilibration period.

For the comparison of DCA and CDCA content in male versus female mouse faecal extracts, an isocratic LC method was used to resolve the closely eluting, isobaric DCA and CDCA peaks. For comparison of male faecal samples to DCA and CDCA standards, the standard samples were prepared by adding 0.8 μl of 5 μg ml^−1^ DCA or CDCA (in 2:1.8 MeOH:H_2_O) to 45 μl of H_2_O. The male faecal extracts were prepared by adding 0.8 μl of 2:1.8 MeOH:H_2_O to 45 μl of extract. All samples were analysed with 30 μl injection volumes. For comparison of female faecal samples to DCA and CDCA standards, the standard samples were prepared by adding 4 μl of 5 μg ml^−1^ DCA or CDCA (in 2:1.8 MeOH:H_2_O) to 20 μl of H_2_O. The female faecal extracts were prepared by adding 4 μl of 2:1.8 MeOH:H_2_O to 20 μl of extract. All samples were analysed with 12 μl injection volumes. Separation was performed on a C18 HPLC column (Zorbax Eclipse Plus, 5 μm particle size, 50 × 2.1 mm). Elution was performed with the same mobile phases as initial female faecal analyses. Phases were delivered at a ratio of 35:65 (mobile phase A to mobile phase B) at a flow rate of 0.7 ml min^−1^.

Eluting species were detected by an Agilent Technologies 6530 Accurate-Mass Q-TOF equipped with an electrospray ion source in negative mode. Ion source settings were: capillary voltage 5,000 V, gas temperature 350 °C, gas flow 12 l min^−1^, nebulizer pressure 40 PSI. For male mouse versus female mouse analysis, the nebulizer pressure was 50 PSI. Analyte identification was performed through comparison of elution time and mass spectra to standard samples. Targets in standards and in extracts were observed as [M−H]^−^ (deprotonated species) and [M+CHOO]^−^ (formate adducts). Data was analysed with MassHunter Qualitative Analysis (Agilent) and custom MATLAB programs.

### *In vivo* exposure to faecal extracts and bile acids and Fos immunostaining

Male B6D2F1/J mice were exposed to faecal extracts and pure bile acids in two separate assays. In the first, 20 mice were placed into a standard mouse cage containing a 10-cm petri dish with a test or control stimulus, and were allowed to freely interact with the stimuli for 10 min. The negative control stimulus was 8–10 g of clean corncob-style bedding (same as the standard bedding on which they were raised) that had been moistened by 1 ml of distilled, filtered water before the test (to match the wetness of test bedding). The positive control stimulus was 8–10 g of corncob bedding that had been soiled by 3–5 BALB/cJ female mice for 48–72 h and frozen at −80 °C until use. All stimuli were dampened with a total volume of 1 ml of water or diluted stimuli 15 min before each test. The two test stimuli were clean bedding to which either 100 μl of pure BALB/cJ female mouse urine or faecal extracts plus 900 μl of water had been added just before the test. Each interaction was video monitored to ensure that each animal interacted with the bedding using its nose during the test period. Following the 10 min interaction period, animals were placed in a cage with clean bedding for 90 min before transcardial perfusion (see below).

In the second test, 10 μl of a control or test stimulus was directly pipetted onto the nares of 15 B6D2F1/J male mice following light isofluorane anaesthesia (5 μl per nostril). The negative control stimulus was 10% methanol (v/v), and test stimuli were 10-fold diluted faecal extract or a mixture of four pure bile acids, CA, DCA, CDCA and LCA, all at 1 mM. Following stimulus exposure, mice were placed onto an empty cage containing only clean bedding for 90 min before transcardial perfusion.

Ninety minutes following free-moving or direct stimulation, animals were anaesthetized by ketamine/xylazine (120 mg kg^−1^ ketamine, 16 mg kg^−1^ xylazine) and perfused transcardially with 4% paraformaldehyde in phosphate-buffered saline (PBS). Brains were extracted and post-fixed in 4% paraformaldehyde in PBS overnight. Following a 3x rinse in PBS, brains were cryoprotected in PBS containing 25% sucrose, then mounted in OCT compound and cut sagittally into 20 μm sections using a cryostat (Leica). Sections were permeabilized with 0.1% Triton-X 100, blocked with 10% goat serum (Sigma), and immunostained with primary antibodies against Fos (Abcam rabbit polyclonal antibody #ab7963, 1:200 dilution), and secondary antibodies conjugated to Alexa Fluor 633 (Thermo Fisher Scientific #A-21070, 1:2000 dilution). Following immunostaining, 3–4 sections from each animal were mounted on slides and counterstained with mounting medium containing DAPI to help identify cell nuclei and the boundaries between AOB sublaminae. Slides were coverslipped and imaged using an AxioScan slide scanner equipped with a 20 ×, 0.8 numerical aperture objective (Zeiss).

Quantification of Fos-staining in AOB sections was performed manually by two observers blinded to the experimental conditions of each section. Boundaries were drawn around the AOB internal and external cellular layers and a curved line was traced along the lateral olfactory tract using basic tools in FIJI/ImageJ. Each observer then counted the number of stained neurons in the AOB, referring to the DAPI nuclear image to avoid double-counting cells. The two observers' scores were strongly positively correlated (data not shown), and the mean of these two observers' scores were used for further analysis. The position of each identified Fos+ cell along the anterior/posterior axis was determined by calculating the shortest distance between the marked cell and the line drawn along the LOT. Differences between experimental conditions were assessed by one-way analysis of variance (total cell density) and Kruskal–Wallis test (anterior-posterior positions).

### Electrophysiology and imaging data analysis

All data analysis were performed in custom software written in MATLAB. Electrophysiological spike responses to stimulus delivery were analysed within a defined 4 s window beginning 1 s after stimulus onset (to allow for complete evacuation of the fluid dead volume within the cannula). The change in spike firing rate (Δ*R*) was determined by subtracting the baseline firing rate before stimulus delivery from the firing rate within the analysis window. Spiking responses were compared with the response to Ringer's control, and significance was evaluated within this window by a two-sample Student's *t*-Test (*P*<0.05 threshold). To be considered for further analysis, the average spiking rate increase in this 4 s window must also have exceeded 1 Hz. Cells that did not demonstrate clearly separable waveforms, either from noise or from other cells, were excluded from analysis. Cells with no discernable stimulus response to any stimulus or that responded to Ringer's control stimuli were discarded. Peristimulus time histograms were generated from the spiking responses within a 20-s window around the stimulus onset (5 s before onset, 15 s after onset). Spiking was evaluated in 1 s bins. Spike rate was averaged across all stimulus repeats to determine the average firing response, which was plotted in a colourised heat map.

Cluster analysis using a modified version of the mean shift method^28^. Briefly, Δ*R* responses on a per-cell basis were normalized to the maximum response across stimuli. Normalized Δ*R* values across cells were passed into the custom clustering algorithm, which compiled a similarity matrix across multiple (typically hundreds to thousands) non-deterministic clustering calculations. The similarity matrix was then analysed by mean shift clustering. This similarity matrix was also displayed in some cases via nonclassical multidimensional scaling (*mdscale* function in MATLAB), which produces 3-dimensional visualizations of the differences between clusters.

To determine the ability of two stimuli to be discriminated from one another, we calculated the discriminability index (*d*^ı^). We calculated *d*^ı^ using the following formula:


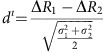


Where Δ*R*_1_ and Δ*R*_2_ represent the mean changes in firing rate to the two stimuli being compared, and 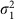 and 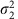 are the variances of Δ*R* across repeated trials. To quantify the significance of the observed *d*^ı^ values, we performed a shuffle test in which 100,000 model populations containing the same number of cells as the observed population were randomly assigned one of the normalized Δ*R* values from each of the compared stimuli. A *d*^ı^ value was calculated for each simulated neuron, and a Kruskal–Wallis test performed between each of the 100,000 randomly shuffled model populations and the observed population. We report percentages of these model populations that achieved statistical significance at the 5% criterion. A higher percentage of statistically significant differences from the model indicates that the observed discriminability was not attributable to random integration of combinations of independent variables, but instead reflected a systematic discrimination between them.

A pairwise comparison matrix was calculated to demonstrate the overlap in responses between bile acids (Fig. 7h). For this calculation, the number of cells that significantly responded to both bile acids was determined, then divided by the number of cells responding to just one of them (the row variable).

In GCaMP3 imaging experiments, changes in fluorescence intensity (ΔF/F) were calculated by dividing the mean pixel intensity for 3 stacks following stimulus by the average baseline intensity in 3 stacks before stimulus delivery. Three-dimensional visualization of the ΔF/F signal was accomplished using MATLAB visualization tools.

### Data availability

Raw data and analysis code are available from the corresponding author on request.

## Additional information

**How to cite this article**: Doyle, W. I. *et al*. Faecal bile acids are natural ligands of the mouse accessory olfactory system. *Nat. Commun.* 7:11936 doi: 10.1038/ncomms11936 (2016).

## Supplementary Material

Supplementary Information Supplementary Figures 1-3
