# Supplementary material for: Faecal bile acids are natural ligands of the mouse accessory olfactory system
Source: Nat Commun. 2016 Jun 21;7:11936. doi: 10.1038/ncomms11936 (PMC4919516; doi:10.1038/ncomms11936)
Supplement: Supplementary Information — Supplementary Figures 1-3 [file ncomms11936-s1.pdf]

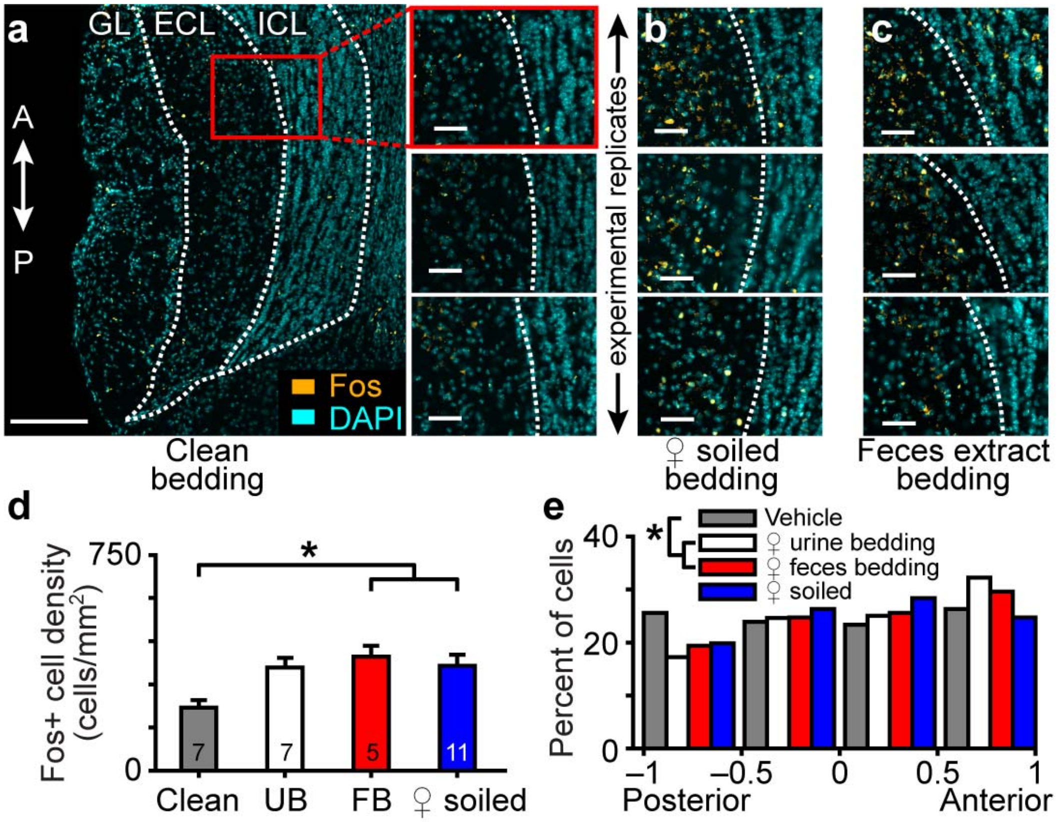

Supplementary Figure 1) Fecal extract activates the AOS *in vivo*. (**a**) Left: Fluorescence micrograph showing Fos immunostaining (orange) in the AOB following 10 minute *in vivo* exposure to clean bedding. DAPI counterstaining shown in cyan. Scale bar 200 µm. GL: glomerular layer. ECL: external cellular layer. ICL: internal cellular layer. A: anterior. P: posterior. Right: zoomed views of the ECL/ICL border for the image shown in Panel a (top) and two other experimental replicates (middle, bottom). Scale bars: 50 µm. (**b-c**) Views of the ECL/ICL border for animals exposed to BALB/cJ female soiled bedding (3 replicates, b) and animals exposed to feces extract-doped bedding (3 replicates, c). Scale bars: 50 µm. (**d**) Total Fos+ neuron density following *in vivo* exposure to clean bedding (gray), urine-doped bedding (UB, white), feces extract-doped bedding (FB, red), and BALB/cJ female soiled bedding (blue) conditions. Asterisk indicates $p < 0.05$ (one-way ANOVA corrected for multiple comparisons). Overlaid numbers indicate replicates. (**e**) Histogram of Fos+ neuron position along the AOB anterior-posterior axis. Asterisk reflects $p < 0.05$ (Wilcoxon rank sum test, cumulative data from 20 animals).

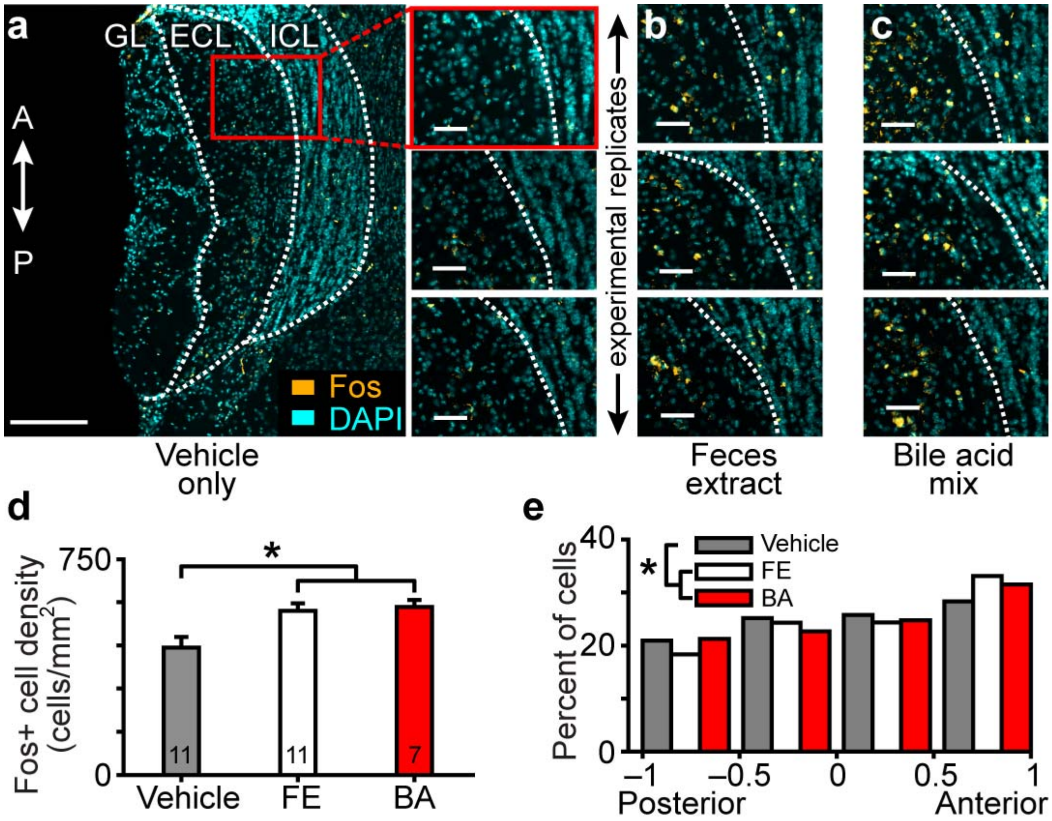

Supplementary Figure 2) Bile acids activate the AOS *in vivo*. (**a**) Left: Fluorescence micrograph showing Fos immunostaining (orange) in the AOB 90 minutes after direct stimulation of the nares with methanol/water (vehicle). DAPI counterstaining shown in cyan. Scale bar 200 µm. GL: glomerular layer. ECL: external cellular layer. ICL: internal cellular layer. A: anterior. P: posterior. Right: zoomed views of the ECL/ICL border for the image shown in Panel a (top) and two other experimental replicates (middle, bottom). Scale bars: 50 µm. (**b-c**) Views of the ECL/ICL border for animals exposed to BALB/cJ female feces extract dissolved in vehicle (3 replicates, b) and animals exposed to a mixture of 4 bile acids (CA, DCA, CDCA, and LCA), each at 1 mM (3 replicates, c). Scale bars: 50 µm. (**d**) Fos+ cell density for direct stimulation conditions. FE: feces extract. BA: bile acid mixture. Asterisk indicates $p < 0.05$ (one-way ANOVA corrected for multiple comparisons). Overlaid numbers indicate replicates. (**e**) Histogram of Fos+ neuron position along the AOB anterior-posterior axis. Asterisk reflects $p < 0.05$ (Wilcoxon rank sum test, cumulative data from 15 animals).

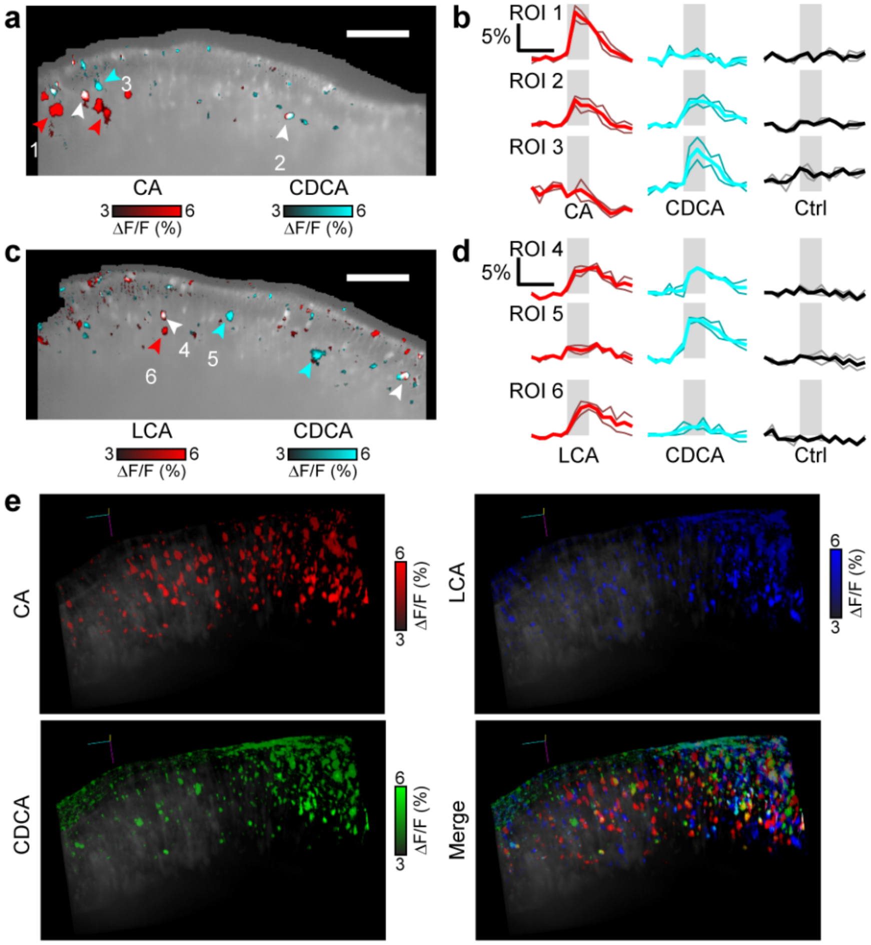

Supplementary Figure 3) Pure bile acids activate VSNs. (**a, c**) Average VSN GCaMP3 responses from 2 individual frames of an image stack spanning 21 x 10^6 μm^3 taken with an objective coupled planar illumination (OCPI) microscope. Grayscale images show basal GCaMP3 fluorescence, and GCaMP3 fluorescence increases during stimulation with 10 μM bile acids are overlaid in cyan and red. Comparisons are between: (**a**) CA (red) and CDCA (cyan), (**c**) LCA (red) and CDCA (cyan). Scale bars: 100 μm. (**b, d**) Normalized fluorescence intensity changes (ΔF/F) for individual ROIs indicated in (a) and (c), respectively. Gray rectangle indicates the 3 image stacks during which stimuli were directly applied to VSNs. (**e**) 3-dimensional VNO activity map showing ΔF/F responses to 3 BAs across a whole image stack. CA (top-left, red), CDCA (bottom-left, green), LCA (top-right, blue). The bottom-right panel shows a merged image. Thin cyan, magenta, and yellow lines: 50 μm scale bars in the x, y, and z directions.
